# Tracking of Mental Workload with a Mobile EEG Sensor

**DOI:** 10.3390/s21155205

**Published:** 2021-07-31

**Authors:** Ekaterina Kutafina, Anne Heiligers, Radomir Popovic, Alexander Brenner, Bernd Hankammer, Stephan M. Jonas, Klaus Mathiak, Jana Zweerings

**Affiliations:** 1 Institute of Medical Informatics, Medical Faculty, RWTH Aachen University, 52074 Aachen, Germany; radomir.popovic@rwth-aachen.de (R.P.); bernd.hankammer@rwth-aachen.de (B.H.); 2 Faculty of Applied Mathematics, AGH University of Science and Technology, 30-059 Krakow, Poland; 3 Department of Psychiatry, Psychotherapy and Psychosomatics, School of Medicine, RWTH Aachen University, 52074 Aachen, Germany; anne.heiligers@rwth-aachen.de (A.H.); kmathiak@ukaachen.de (K.M.); jzweerings@ukaachen.de (J.Z.); 4 Institute of Medical Informatics, University of Münster, 48149 Münster, Germany; alexander.brenner@uni-muenster.de; 5 Department of Informatics, Technical University of Munich, 85748 Garching, Germany; jonas@in.tum.de

**Keywords:** mHealth, EEG, N-back, cognitive effort, wearable

## Abstract

The aim of the present investigation was to assess if a mobile electroencephalography (EEG) setup can be used to track mental workload, which is an important aspect of learning performance and motivation and may thus represent a valuable source of information in the evaluation of cognitive training approaches. Twenty five healthy subjects performed a three-level N-back test using a fully mobile setup including tablet-based presentation of the task and EEG data collection with a self-mounted mobile EEG device at two assessment time points. A two-fold analysis approach was chosen including a standard analysis of variance and an artificial neural network to distinguish the levels of cognitive load. Our findings indicate that the setup is feasible for detecting changes in cognitive load, as reflected by alterations across lobes in different frequency bands. In particular, we observed a decrease of occipital alpha and an increase in frontal, parietal and occipital theta with increasing cognitive load. The most distinct levels of cognitive load could be discriminated by the integrated machine learning models with an accuracy of 86%.

## 1. Introduction

Research and technological advances of the last several decades have led to an increased awareness of the importance of personalized approaches to human medical care and wellbeing. In the domain of mental health, data-driven individualized feedback is cautiously being integrated to support diagnosis and the development of personal treatment schemes. Mental health apps are a promising approach to collecting data at several time points with minimal distortion of the everyday life routines of the participant. However, empirical support is often lacking (see review [1]) as many applications are limited to self-reported data. While such data is an important source of information, it may be distorted by interoceptive ability and social desirability biases. In addition, many psychiatric disorders are underpinned by alterations on the neural level that cannot be captured by self-reporting measures but may represent a valuable source of information for the development and evaluation of treatment schemes. For example, electrophysiological data such as electroencephalography (EEG) and electrocardiography (ECG) have been used to generate information on the emotional state [2] or the stress level of an individual [3] as well as for diagnostic purposes and the evaluation of treatment progression [4].

In particular, EEG measurements are a promising indicator of mental workload [5] which is a general term describing the relationship between the task demands and mental resources needed to be employed to complete a task. The actual workload is related to several aspects including task demands and working memory capacity. In an attempt to classify workload, Hogervorst and colleagues [5] compared the performance of several physiological markers, including skin conductance, respiration, ECG, pupil size, eye blinks, and EEG while inducing different levels of workload with an N-back task. According to the authors, classification of workload was best for the EEG data. Importantly, the performance of the classification did not significantly improve upon combining different features. In previous studies, cognitive load has been associated with variations in different EEG frequency bands. Most consistently, changes in the alpha and theta frequency bands have been reported in response to different levels of workload [6,7,8]. For example, alpha power has been shown to increase during rest, i.e., when mental load is low [6]. Upon intensification of mental workload, alpha power has been shown to decrease [9]. The opposite response pattern can be observed for theta frequency bands, which show an increase with rising mental effort [10].

Thus, EEG-informed assessment of mental workload may provide a feasible solution for the objective evaluation of task difficulty. This is highly relevant to many practical applications, including safety assessment in the context of driver or pilot fatigue [11,12] and working efficiency or learning [13]. In a clinical context, objective measures of workload could access the success of cognitive trainings, which are promising for the betterment of brain injury or stroke survivors [14,15], as well as for patients with mental disorders [16]. An objective and easy to measure physiological marker for brain function improvement could support clinical decision making by providing additional information on the efficacy of treatment options and hence, information that is important in the context of the start or termination of the therapy. In addition, deriving objective markers for cognitive load could be used to develop adaptive training protocols, thereby increasing training efficiency and creating individually tailored training protocols without the necessity for human monitoring. This is important since two essential problems are associated with self-monitoring of cognitive load. Firstly, the user needs to have an adequate interoceptive awareness of the mental effort needed for the task. Secondly, the monitoring necessitates a dual-task performance (i.e., completing the task and monitoring cognitive workload) that will require additional cognitive resources and hence may reduce task performance and learning [13]. Flexible adjustment of the training based on an objective marker of mental effort may enhance training performance by preventing excessive demands on the patients that may decrease motivation.

So far, research has mostly implemented clinical or research-grade EEG devices to measure brain function in healthy individuals and patients. While these devices offer advantages in terms of high signal quality, their setup is time consuming and cannot be used in home-care scenarios. To realize an implementation of personalized care in daily medical routines that extends beyond health care in hospitals, facilitation of use of the devices, an optimal cost-benefit association, and time-efficiency are essential. The development of mobile sensors provides the possibility to perform measurements quickly, easily, and without the necessity of visiting a medical facility, thus promoting the expansion of health-care options in the natural settings of the patients. For example, Hussain and Park [17] proposed a set-up with mobile EEG built into a sleeping mask for ischemic stroke patients’ monitoring, employing machine learning algorithms for group classification. In another work, Murphy et al. [18] used machine-learning on dry-electrode EEG data to track cognitive aging.

In this project, we aimed to determine the potential of a mobile EEG device to detect changes in electrical brain activity based on cognitive load. In order to achieve this goal, we implemented a fully mobile setup consisting of a mobile EEG device connected to a tablet to execute the cognitive performance task. In line with previous investigations, we expected to observe frequency band changes, particularly a reduction in alpha and an increase in theta band power depending on cognitive load, as measured by a modified version of the N-back task. To create a comprehensive display of the data, we combined a statistical approach with a machine learning-based condition classification. Furthermore, we wanted to assess the usability of the setup by the participants to evaluate if its self-responsible use in daily life is feasible.

The current investigation contributes to our understanding of the applicability of mobile EEG devices in the context of the assessment of cognitive load. This aspect is of high importance since it may serve as a starting point for various applications in daily life such as the development of an assistive tool for cognitive trainings in patients to monitor and flexibly adapt the difficulty level of the training. In the following, we will describe the experimental set-up (tasks, hard- and software), the two-fold analysis approach (standard analysis of variance and the artificial neural network), and the findings on the feasibility of our set-up and user experience.

## 2. Materials and Methods

### 2.1. Participants

Twenty-five healthy volunteers participated in this monocentric prospective study. One participant was excluded from data analysis based on the results of the clinical interview resulting in a final sample for data analysis of 24 (age in years: 24.4 ± 3.3; 17 female; see Table 1). All participants were recruited by advertisement at the RWTH Aachen University Hospital, native German speakers and right-handed according to the Edinburgh Handedness Inventory with the exception of one individual. All participants had normal or corrected-to-normal vision. Inclusion criteria were age between 18–65 years, voluntary participation and ability to consent; severe psychiatric disorders and epilepsy served as exclusion criteria. To objectify the criterion “severe psychiatric disorder” the screening of the German version of the Structured Clinical Interview for assessment of DSM-IV-TR criteria [19] was performed by medically trained personnel. According to the interview, participants had no history of or current psychiatric disorder. All participants gave their written informed consent prior to inclusion in the study and received 20 € as compensation for their participation. The study was submitted to and approved by the local ethics committee of the Faculty of Medicine of the RWTH Aachen University (EK 284/18). The pre-registration at the German clinical trial register (Deutsches Register klinischer Studien, DRKS00016150) comprised the hypothesized theta and alpha effects as primary outcome. Analysis of the P300 component will be reported at a later time point.

Prior to the start of the main study, the technical setup was tested on the researchers to prevent unexpected failure of hardware or software components as well as to check the overall experimental flow.

### 2.2. Experimental Setup

#### 2.2.1. Experimental Procedure

**Figure 1:**
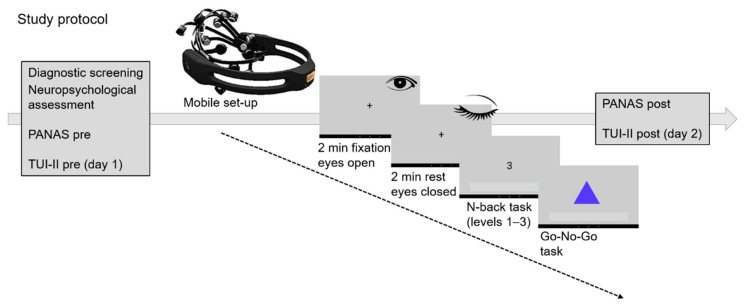
Study protocol. Participants completed two lab visits. On day 1 they received a diagnostic screening and neuropsychological assessment, and had to fill in the pre-assessment of the usability questionnaire. On day 1 and day 2, participants completed the PANAS to assess the affective state prior to and after the EEG recordings. Prior to the start of the cognitive tasks, participants received standardized instructions for the setup of the mobile EEG device. After successful application, a fixation cross was displayed and participants were instructed to keep eyes open, followed by two minutes of rest with eyes closed. Afterwards, participants completed the N-back task with three difficulty levels (presented in randomized order) and the Go/No-go task. On day 2 only, participants filled in the TUI-II-post questionnaire and the System Usability Scale after completion of the tasks.

Participants were invited to the lab on two days (see Figure 1). The two measurement days were separated by at least one week, but not more than two weeks. Measurements took place at the same time of day for each participant individually; however, measurement times between participants varied. On day 1, the diagnostic screening took place and sociodemographic data was acquired. All participants completed two neuropsychological measures: a verbal intelligence test (Wortschatztest—WST, [20]) and a working memory test (digit-span task). Furthermore, the German version of the Positive and Negative Affect Scale (PANAS, [21]) was administered to assess the affective state of the participants. In order to investigate the usability of the device, participants had to complete the Technology Usage Inventory (TUI-II, [22]). On day 1, two subscales of this inventory were selected: (a) technology anxiety and (b) curiosity. After completion of the questionnaires, all participants received a verbal explanation on the setup of the mobile EEG device. Subsequently, subjects were asked to independently use the device under the guidance of a simplistic user manual with illustrations of the application of the device. At this stage of the experiment, participants received help from the investigator if they were not able to place the electrodes adequately themselves to ensure a sufficient signal-to-noise ratio.

The EEG measurements started with a baseline recording which included fixating on a cross for 2 min, resting with closed eyes for 2 min, and tapping on the screen ten times. After the baseline measurement, the N-back task was performed, followed by a Go/No-go task. Analysis of the data from the Go/No-go task was not in the focus of the current manuscript. Prior to starting the Go/No-go task, the investigator ensured that the electrode setup was still functional. If a malfunction was detected, the EEG setup was corrected. After the EEG measurement the PANAS was administered a second time to evaluate changes in the affective state. In line with the work of Brouwer and colleagues [7], the current investigation focuses on data of the N-back task as a measure of cognitive load and data quality only.

Day 1 and day 2 were constructed in exactly the same way with exception to the questionnaires. On day 2, only the PANAS questionnaire was administered prior to and after the measurement as well as the post evaluation of the TUI-II including the subscales ‘skepticism’, ‘interest’, ‘accessibility’, ‘user-friendliness’, and ‘usefulness’. In addition, all subjects completed the System Usability Scale (SUS, [23]).

#### 2.2.2. Hardware

**Figure 2:**
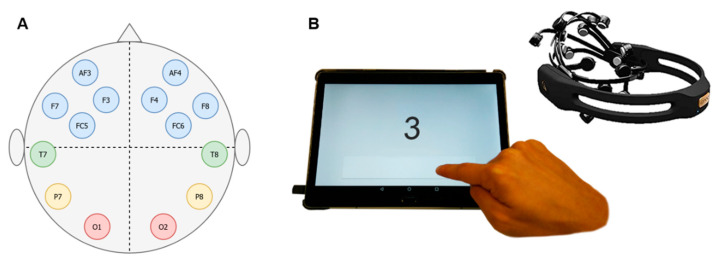
(**A**) Electrode placement. The figure depicts electrodes that are covered by the Emotiv Epoc mobile EEG device. Frontal electrodes are depicted in blue (*n* = 8), temporal electrodes in green (*n* = 2), parietal electrodes in yellow (*n* = 2) and occipital electrodes in red (*n* = 2). (**B**) Mobile setup. Display of the mobile EEG device and the tablet that was used for stimulus presentation. The figure illustrates an example of the N-back task. Participants were instructed to press the ‘button’ (visible on the lower part of the tablet screen) upon detection of a target stimulus. The rectangular button covered an area of 172 mm × 20 mm.

In the present study, we used a consumer grade EEG device, namely the Emotiv Epoc+ with sampling rate of 128 Hz (EMOTIV Inc., San Francisco, CA, USA). One of the reasons to use this device is its relatively large brain area coverage (14 electrodes, located bilaterally over the occipital lobe (2), temporal lobes (2), parietal lobe (2), and frontal lobe (8); see Figure 2A). It is important to note that the rigid plastic frame determines the position of the individual electrodes. This is compensated by the fact that the saline solution-based electrodes and wireless construction allow for a very fast and easy mounting, which can be used independently by participants who are naive to the method. Importantly, the Emotiv Epoc has been validated for a variety of research purposes [24], including an investigation on simultaneous collection of mobile EEG and clinical EEG on patients with epilepsy. Results of those studies indicate lower signal-to-noise ratio in the data from the mobile device compared to clinical-grade devices, and limited brain coverage [25]. However, the quality of the data from the Emotiv Epoc recordings had adequate quality to track epileptiform discharges [26], specific ERP (event-related potentials) changes [24,25], and frequency domain fluctuations [27], rendering it a promising candidate for the current investigation.

The mobile EEG device was connected to an Android tablet (Sony Xperia Z2) via Bluetooth. Since the primary goal was to create a fully mobile setup, we decided to use a tablet as a display medium. In addition, the tablet is beneficial in terms of the facilitation of use. We implemented simplistic graphic design choices that allow for an easy navigation through the experiments and control via tapping on the screen. To ensure similar distances to the display screen across participants and fast reactions, the tablet was placed on a bracket. The screen of the tablet had a size of 295.8 cm^2^ with a resolution of 1920 × 1200 pixels (16:10 ratio, ~224 ppi density).

#### 2.2.3. Software

A custom application developed for Android 6.0 API 23 (Marshmallow) was used for the connection to the mobile EEG. Data recording was performed in the background while the experiment interface was presented. Experiment data, such as stimuli types and task descriptions, were read from an XML format. During experiments, tasks were presented visually and button presses were recorded. The area that was sensitive to the ‘button press’ of the participants was defined as a rectangular Android button of size 172 mm × 20 mm. Visualization of the “pressed button” served as a feedback to the subject to avoid multiple responses to a single stimulus. Event information in discrete time steps were stored in the European Data Format (EDF) with a resolution of 128 Hz (one sample equals 7.81 ms). With a stimulus onset interval of 1600 ms, the actual measured time between stimuli was on average 1599.36 ms with a standard deviation of 9.74 ms. Response times were recorded with the timestamp provided by the Android system’s clock. Impedance values were regularly collected during the recording for each channel and added to the EDF data files.

#### 2.2.4. N-Back Task

**Figure 3:**
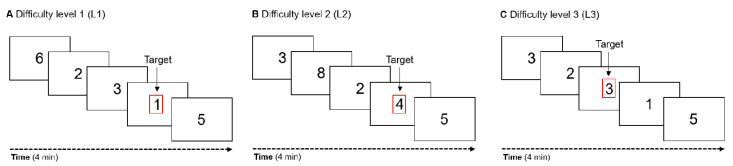
Illustration of the three difficulty levels of the N-back task. Participants were instructed to press a button as fast as possible whenever a target appeared. Each condition contained 150 stimuli (50 targets) that appeared on the screen for 1600 ms each. (**A**) Participants were instructed to press a button whenever the digit ‘1’ appeared on the screen. (**B**) Participants had to press a button when three digits in a row were even. (**C**) Participants were asked to press a button when the digit was identical to the second to last digit.

An N-back task with three difficulty levels but identical stimuli and target rates was implemented to manipulate cognitive load (see Figure 3). The task design was adapted from Mathiak and colleagues [28]: A sequence of 150 black digits (font: Roboto, height: 16.2 mm) was presented to the participants in each condition on a grey background in full screen mode (Figure 2B). One third of these digits (*n* = 50) were targets in each condition. The presented digits (1 through 8) were shown for 1600 ms each, and immediately followed by the next digit resulting in a total duration of 4 min for each condition. Participants were instructed to respond as fast and accurately as possible. In the first condition ‘zero-back’ (difficulty level 1, L1) participants had to press a button each time the digit “1” was shown. In the second condition ‘three-even’ (difficulty level 2, L2) participants were instructed to press a button whenever three consecutive digits were even. In the third condition ‘two-back’ (difficulty level 3, L3) the participants were asked to press a button whenever the presented digit was identical to the second to last digit.

Two independently pseudo-randomized versions of each condition were created (version A & B). The versions were constructed in a parallel way, i.e., the same amount of stimuli and targets. However, the randomization of digits differed between conditions. Randomization of the digits was performed with the constraint that the same number is not shown twice in a row and with a defined target rate of 33%. Half of the participants completed version A on day 1 and version B on day 2, and the other half completed both versions in reversed order. Furthermore, the order in which the three conditions were presented was randomized for each participant. At the beginning of each condition the participants were asked to focus on a fixation cross presented in the middle of the screen for 1 min. This period formed the baseline for EEG analyzes (L0).

### 2.3. Data Analysis

#### 2.3.1. Software

Data preprocessing and feature extraction was performed using MATLAB 2019a (The Mathworks, Natick, MA, USA) and EEGLAB 2019 [29]. Statistical analysis was conducted using the Statistical Package for the Social Science (SPSS) software, version 25 (IBM, Armonk, NY, USA).

#### 2.3.2. Behavioral Data

To validate the difficulty levels of the N-back task in the present sample, average response times for each condition across days were calculated and subjected to repeated measures ANOVA with the within-subject factor condition [L1, L2, L3]. Paired sample t-tests were carried out for post hoc evaluations. To further quantify the performance and minimize biases due to non-adherence to task instructions (e.g., randomly pressing the button), we calculated the ‘fraction correct’ as a measure of accuracy such as proposed by Brouwer and colleagues [7] as the number of correct responses divided by the total number of stimuli. In parallel to the reaction time task, the ‘fraction correct’ data was subjected to a repeated-measures ANOVA with the within-subjects factor condition [L1, L2, L3].

To assess usability of the setup, this study applied the SUS; the composite score was established according to [23] by calculating the sum of the scores for each individual item and multiplying the sum by 2.5. Items were rated on a five-point Likert scale. This procedure results in a composite score ranging between 0 and 100. In addition, the composite score of the TUI-II was used to evaluate usability of the device [22]. Items were rated on a seven-point Likert scale.

#### 2.3.3. EEG Data Preprocessing

**Figure 4:**
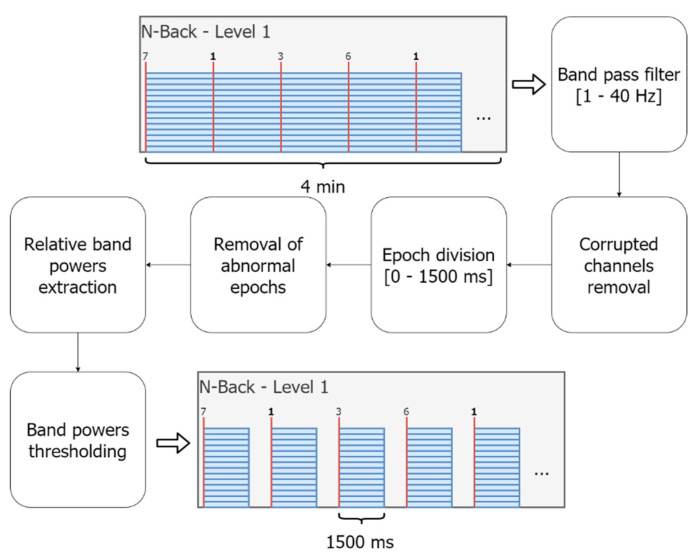
The continuous EEG data were preprocessed (band pass filtered at 1–40 Hz, channel rejection) and split into event-related epochs of 1500 ms each. After epoch rejection, powers of the individual frequency bands (delta-beta) were extracted.

The implemented preprocessing pipeline can be separated into six consecutive steps (see Figure 4): (1) band pass filtering, (2) automatic channel removal, (3) epoch extraction, (4) automatic epoch removal, (5) extraction of scaled power for each frequency band, and (6) rejection of outliers in band power. Mobile EEG has been shown to have a lower signal-to-noise ratio compared to clinical grade devices [26], and therefore it is particularly important to track the amount of the removed data. Accordingly, we collected the respective statistics on each preprocessing step.

1. Filtering. Previous research [27] established optimized frequency filtering for the Emotiv Epoc at a 128 Hz sampling rate including a high-pass filter at 1 Hz and a low-pass filter at 40 Hz. Implementing of high-pass filtering as a first step in the preprocessing pipeline is of particular importance, as the unfiltered slow drift is heavily represented in the data. The chosen level of the low-pass filter ensures the removal of the power line.
2. Corrupted channels removal. The standard EEGLAB function (pop_rejchan) was used to remove the complete corrupted channels based on their normalized kurtosis.
3. Epoch extraction. The filtered data was divided into epochs, covering a 1500 ms period after the stimulus presentation [30]. The resting state data, recorded during a fixation cross presentation on the screen while participants were assigned no specific task, were cut into 1500 ms segments and served as a baseline condition. The following information was additionally stored for each epoch: (1) target or non-target stimulus and (2) response exhibition prior to the next stimulus display.
4. Removal of abnormal epochs. EEGLAB kurtosis-based deviation detection function was used to remove epochs with artefacts. The epochs with an absolute amplitude above 1500 or impedance below 100 (internal device-specific units) were removed subsequently.
5. Relative band powers extraction. Power measures for the EEG bands were extracted for each epoch using the following boundaries [31]: delta (1–3.5 Hz), theta (3.5–7.5 Hz), alpha (7.5–12.5 Hz), and beta (12.5–30 Hz). The resulting values were further logarithmically scaled [32] to improve the power distributions across the bands and normalized by the similarly scaled and averaged full band (1–40 Hz) power during the baseline condition L0. Normalization was performed separately for each participant and each day.
6. Band power threshold. Epochs with any relative frequency band value exceeding the threshold of 1.3 were removed.

In this work, we did not specifically handle EMG (electromyography), ECG (electrocardiography), or EOG (electrooculography) artifacts. The most prominent contaminations caused by those artifacts are removed through frequency filtering, removal of abnormal epochs, or by power band threshold.

#### 2.3.4. Statistical Analysis

The statistical analysis was performed to investigate differences in the logarithmic band power between conditions, taking into account the inter-personal and inter-session variations. The preprocessed data was averaged across records to prevent artificially improved results due to the high number of epochs within the same (subject, day, condition, electrode) combination, which cannot be treated as independent entries. In addition, the data was averaged across the brain lobes (see Figure 2A). The EEG data was analyzed using a repeated measures ANOVA with the within-subject factors condition [L0, L1, L2, L3], where L0 reflects the baseline, Frequency Band [delta, theta, alpha, beta], Lobe [Frontal, Parietal, Temporal, Occipital], and Day [Day 1, Day 2]. Our results are obtained on the data which includes all epochs, without separation into the target versus nontarget stimuli or response correctness.

#### 2.3.5. Machine Learning

In this paper, we applied artificial neural networks (ANN) to classify the experimental conditions. The ANN approach proved to be efficient in EEG applications, in particular, when combined with spectral features such as frequency bands or wavelets [33]. Standard feed-forward shallow networks, as implemented in MATLAB 2019a, were used. The classification solution can be presented as three steps: (1) data division, (2) optimization of the ANN architecture, and (3) averaging across randomizations to improve model robustness. This separation should highlight the high number of degrees of freedom which needed to be addressed before the final parameter choices were made.

**Figure 5:**
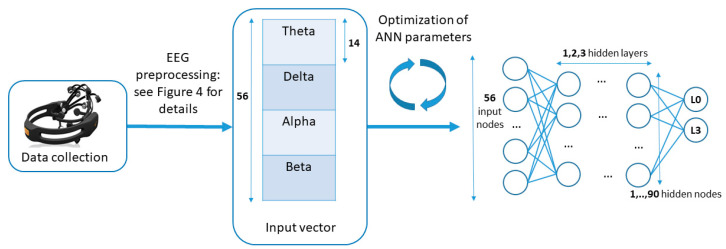
After preprocessing, the EEG data vector (input) has length 56 (4 bands × 14 channels). Correspondingly, the number of ANN input nodes is 56, the output layer during the optimization process has 2 nodes (we classify only L0 and L3 difficulty levels). The number of hidden layers vary between 1 and 3, and the number of hidden nodes is kept the same for all hidden layers and takes values 1, 5, 10, 15, 20, 30, 40, 50, 60, 70, 80, 90.

1. Train/test data division. We expected high data variation in this data set. Therefore, we tested four different approaches regarding the data division into test and training sets. Firstly, participants were randomly divided into three groups of 16, 4, and 4 individuals each and the corresponding data was used as training, validation, and test sets, respectively. The validation set served parameter adjustment purposes to avoid that the parameters are optimized to perform best on the test set only. Subject-wise data division is the most desirable for the potential applications as it allows us to work with the new participants’ data without personal calibration. Secondly, all data from day 1 was used as a training set, the first half of the day 2 data as a validation set, and the second half of day 2 as a test set. This approach reflects a situation in which personal calibration is required, but further new input from the same person can be analyzed directly. Next, all data from day 1 and first half of the day 2 data served as a training test. The last part of day 2 was divided into 2 equal parts for validation and test. Lastly, a leave-one-out approach was explored once the parameter optimization was finished. This allowed us to use more data for the training of the model and to investigate how promising it would be to skip personal calibration if more training data becomes available.
2. Optimization of the ANN architecture (see Figure 5). Our initial input vector size equals to 4 (number of the used frequency bands) × 14 (number of channels) = 56. We performed a consecutive search through the combinations of 1 to 3 layers as well as with 1, 5, 10, 15, 20, 30, 40, 50, 60, 70, 80, and 90 nodes (same for each layer). The network output was a vector of size 2, which assigned the input data to one of the two classes. By default those classes were difficulty level 0 (L0) and 3 (L3). The network architecture with the best accuracy in differentiation between those two classes was chosen for further experiments. It is important to note that the data was being permuted before entering the ANN structure in order to optimize the training process.
3. Averaging across multiple randomizations. To control for the effect of the initial weight randomization of ANNs, training in every architecture setup step was repeated 30 times and the results were averaged. After the optimal network size was fixed, 10 instances of the ANN were trained on the train set to create a voting system. This way, potentially large fluctuations of the results due to the initial weight randomization were smoothened to obtain more robust results.

## 3. Results

### 3.1. Behavioral Data

#### 3.1.1. Reaction Time Data

**Figure 6:**
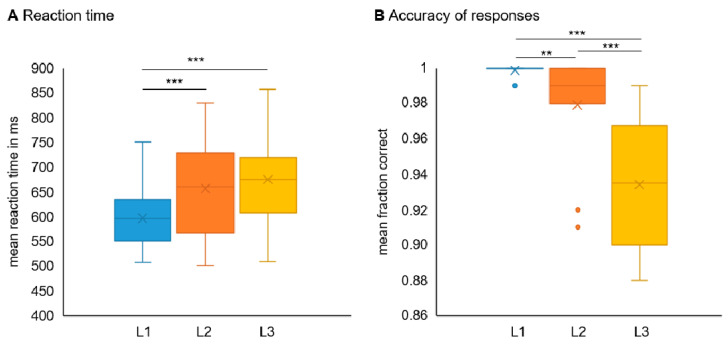
The behavioral analysis confirmed the experimental conditions as reflected by increasing task demand with slower responses and reduced accuracy across the three conditions (corresponding to difficulty levels L1, L2, L3). (**A**) Mean reaction times across days for each condition. A significant main effect of condition emerged. Post hoc tests indicate a significant difference between L1 and L2 and L1 and L3. *** *p* = 0.001. (**B**) Mean fraction of correct responses across days for each condition. A significant main effect of condition emerged. Post hoc tests indicate a significant difference between all conditions. *** *p* = 0.001, ** *p* = 0.005.

The repeated measures ANOVA with the factor Condition [L1, L2, L3] revealed a significant main effect (*F*(2,46) = 12.78, *p* < 0.001, η_p_^2^ = 0.357; see Figure 6). Post hoc paired *t*-test for each pair of conditions (i.e., L1 vs. L2, L1 vs. L3, L2 vs. L3) were calculated. Results indicate a significant difference between L1 and L2 (*t*(23) = −3.95, *p* = 0.001) and L1 and L3 (*t*(23) = −5.22, *p* < 0.001). No significant difference was detected between L2 and L3 (*t*(23) = −1.01, *p* > 0.2). On average, reaction times were 596.9 ms (±59.4 ms) for condition 1, 656.9 ms (±95.6 ms) for condition 2, and 675.4 ms (±59.4 ms) for condition 3.

#### 3.1.2. Accuracy of Responses

The ‘fraction correct’ (FC; i.e., the number of correct responses in comparison to all responses) was calculated across days and entered repeated measures ANOVA with the factor condition. The main effect was significant (*F*(2,46) = 41.26, *p* < 0.001, ηp^2^ = 0.642). Post hoc paired samples t-tests revealed a significant difference between all conditions (L1 and L2: *t*(23) = 3.23, *p* < 0.005; L1 and L3: *t*(23) = 8.97, *p* < 0.001; L2 and L3: t(23) = 5.31, *p* < 0.001). FC decreased across conditions—and hence with an increase in cognitive load —with a mean FC of 0.998 (±0.003) in condition 1 (L1), 0.98 (±0.03) in condition 2 (L2) and 0.93 (±0.03) in condition 3 (L3). Accordingly, all participants performed above chance level with FC scores of 0.88 and higher. The distribution of the data was skewed to the left—in particular for L1. According to the Shapiro-Wilk test, the assumption of normality was violated. However, based on a sufficiently large sample size and the relative robustness of the repeated measures ANOVA and t-statistics against non-normality if the other assumptions are met, we decided to report the results [34,35,36]. Four data points can be considered outliers in L2 based on a criterion of ±3*SD*. These data points represented individuals with a higher number of mistakes across conditions. Since the FC scores of the respective individuals were still clearly above chance, we did not exclude them from analysis. In addition, test-statistics were not heavily influenced by these values.

#### 3.1.3. Usability

In the current investigation, participants indicated on average a SUS composite score of 79.6 (±11.2) indicating subjectively perceived good usability [37]. Notably, more than 70% of the participants had a score higher than 80% (*n* = 17).

According to the TUI-II, participants had low levels of anxiety with regard to testing new technology (technology anxiety: 1.7 ± 0.63) and were curious to test it (curiosity: 4.2 ± 1.3) prior to the training. Upon completion of the training, participants indicated that they have little skepticism with regard to the setup (2.1 ± 0.7) and had medium levels of interest (5.2 ± 0.9), accessibility (4.5 ± 0.9), and usefulness (4.5 ± 1.0). The highest ratings were obtained for user-friendliness (5.9 ± 0.9), indicating that participants experienced the use of the mobile EEG setup as convenient.

### 3.2. EEG Data

#### 3.2.1. Statistical Results

**Figure 7:**
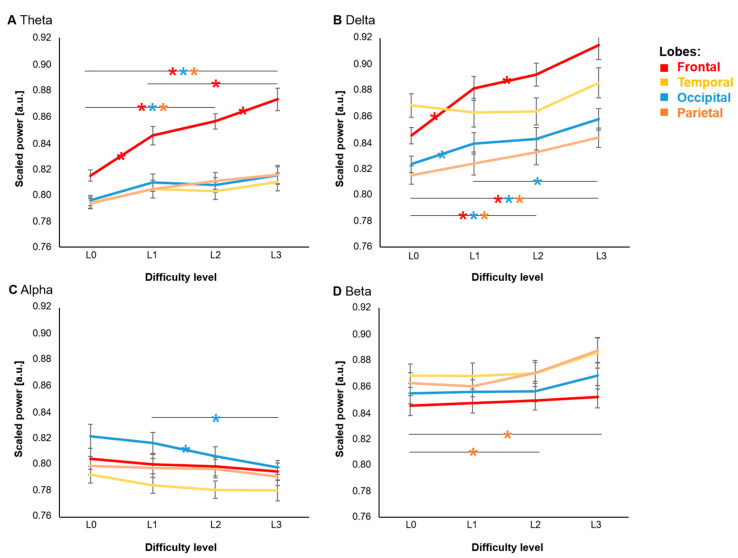
Marginal means with confidence intervals in three-factor ANOVA of power measures. The panels illustrate the logarithmically scaled power measure in the different EEG frequency bands (**A**): theta, (**B**): delta, (**C**): alpha, (**D**): beta. On the *x*-axis, the different conditions are mapped with increasing cognitive load (L0: baseline; L1: detection; L2: one-back; L3: 2-back). Data are averaged for electrodes over the four cerebral lobes (red: frontal; yellow: temporal; cyan: occipital; orange: parietal). Stars in the same color highlight significant post hoc comparisons across conditions for the respective lobe (e.g., a blue star on the line between L1 and L2 indicates a significant difference between these conditions for the frontal lobe with * *p* < 0.01). The robust replication of the primary hypotheses using the mobile technology can be observed as: increase of frontal theta (red curve and stars in Panel A) and occipital alpha (blue in Panel C) with increasing cognitive load (from L0 to L4) decreased with higher cognitive load. Further, a similar pattern emerges for the delta band and potentially the beta band as well. * *p* < 0.01, a.u.: arbitrary unit.

Results of the repeated measures ANOVA were organized by lobes to increase comprehensibility of the data. This approach was chosen due to high intra-lobe consistency of the data. Importantly, eight electrodes represent the frontal lobes, whereas the parietal, temporal, and occipital lobes are represented by two electrodes each. The repeated measures ANOVA revealed a significant three-way interaction between Lobe, Band, and Condition (*F*(27,594) = 14.42, *p* < 0.001) indicating band-specific effects. In addition, significant main effects of Band (*F*(3,66) = 19.28, *p* < 0.001), Lobe (*F*(3,66) = 17.59, *p* < 0.001) and Condition (*F*(3,66) = 5.15, *p* < 0.005) emerged. No significant effect for Day was found (*F*(1,22) = 0.49, *p* > 0.4). To increase comprehensibility of the results, we grouped the findings by frequency bands (see Figure 7 and Table A1 in the Appendix A).

Post hoc t-tests confirmed an increase of frontal theta relative band power for all comparisons between conditions apart from L1L2 (*p* < 0.01). For occipital and parietal theta, comparisons between baseline L0 and L2/L3 revealed significant differences (*p* < 0.01). In addition, occipital alpha relative band power was significantly reduced for the comparisons between L1 and L2/L3 (*p* < 0.01).

In addition, we explored the relative power in beta and delta frequency bands. Significant differences emerged for frontal delta (all comparisons apart from L2–L3; *p* < 0.01) and occipital delta (all comparisons apart from L1–L2 and L2–L3; *p* < 0.01). For parietal delta only the comparison between baseline L0 and L2/L3 revealed significant differences (*p* < 0.01). Overall, relative delta frequency band power increased with task difficulty. For parietal beta, significant differences were found for L0–L3 and L1–L3 (*p* < *0*.01; see Table A1 in the Appendix A), reflecting an increase of relative band power with task difficulty.

In summary, we could confirm the pre-registered primary endpoints in terms of a reduction of occipital alpha and an increase of frontal theta with increasing cognitive load. Effects similar to the theta band emerged in the delta and—to a lesser extent—in the beta frequency domains.

#### 3.2.2. Classification of the Conditions with Machine Learning

##### ANN Optimization for Different Data Division Setups

To investigate the fitting properties of ANN, we tested different network architectures (number of layers and nodes) for 56 features (4 bands, 14 electrodes) and the 3 different options of the data division (random participant assignment, first day only chosen as a train set, half of the second day added to the train set). Only very small differences emerged between different architectures, which may be attributed to efficient internal MATLAB optimization algorithms. The differences between versions of train and test data separation were more substantial and in line with our expectations. Namely, the optimal result in accuracy (86.24%) was achieved using 1.5 days of the experiment as a train set for the remaining 0.5 day as test set.

##### Performance in Different Recognized Classes

**Figure 8:**
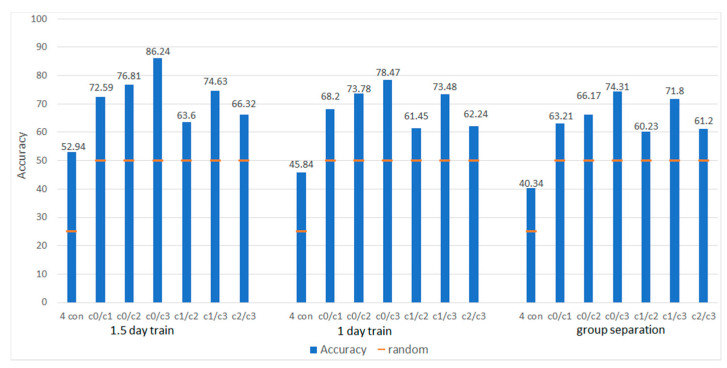
Accuracy of the ANN classifier for different train/test data divisions (training data from 1.5 day, training data from 1 day, training and validation data from separate subjects). The accuracy is shown for the separation of all four conditions (25% chance level, orange bar) and between each pair of conditions (50%). The classification accuracy profits from using data from the same day (1.5 day condition) and from the same subject (1 day condition) as well as from a stronger difference between the cognitive load conditions (e.g., L0–L3 versus L1–L2). This pattern suggests that inter- and intra-subject variance affect the performance but also that the network seem to rely on relevant cues since the separation is improving with the load difference.

**Figure 9:**
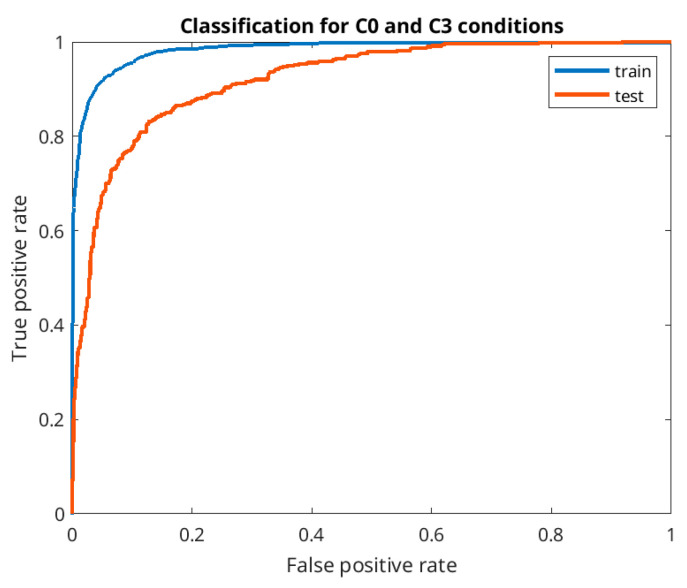
ROC (receiver operating characteristic) curves for two-classes discrimination for the train (blue) and test (red) data. AUC (area under the curve) is correspondingly 0.98 and 0.92 for the train and test data.

Applying the optimal network architecture for each train/test separation, we investigated the pairwise separation of the conditions: L0 vs. L1, L0 vs. L2, L0 vs. L3, L1 vs. L2, and L2 vs. L3. Additionally, we increased the number of ANN output classes to four (the rest of the architecture remains the same), and classified all four conditions simultaneously. The average accuracy is displayed in Figure 8 in reference to chance level (orange lines at 25% for all four and 50% for two conditions). The highest accuracy was obtained for L0 and L3 after training with 1.5 day data (86.24%). For better illustration, the receiver operating characteristic curves (ROC) for train and test data are presented on Figure 9. Sensitivity and specificity of the test data are well-balanced with the values 0.83 and 0.86 correspondingly, precision of 0.78 and recall of 0.83. This result confirmed that the most discriminative workload levels led to the highest difference in the separating features. The lowest accuracy of 40% emerged in classification of all four workload levels with randomized individuals, still remaining above chance level. The same four-condition discrimination accuracy improved to 45% when training data came from the same individuals but another day and to 53% using within-session calibration (1.5–0.5 day data split).

**Figure 10:**
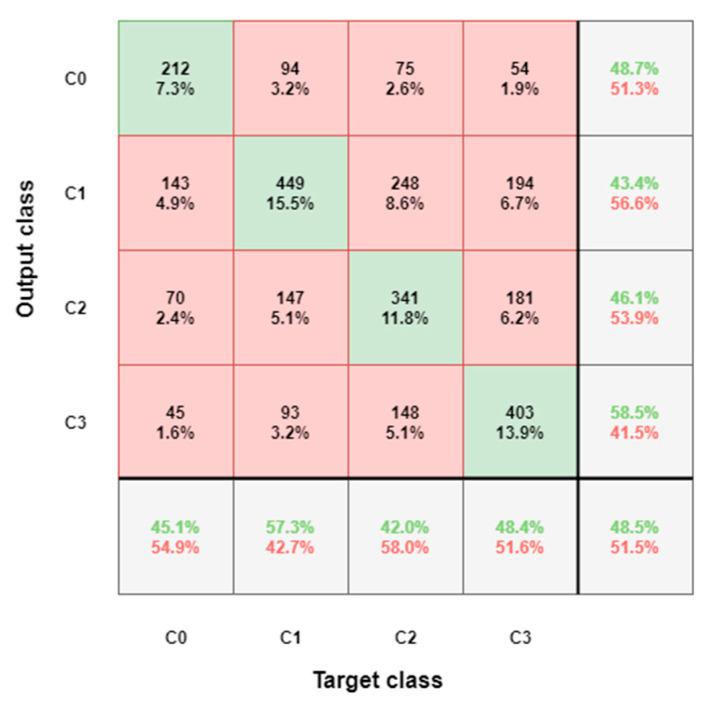
Confusion matrix for four conditions discrimination (classes 0, 1, 2, 3). Overall, an accuracy of 48.4% almost doubles the random guess baseline (25%) and the confusion between neighboring classes is higher than between more distinct ones.

The confusion matrix for the four conditions’ classification reveals that the errors are rather evenly distributed and the closer together the conditions are, the higher the chance of confusions (Figure 10).

##### Data Subsets: Target/Non-Target, Correct/Incorrect

It can be expected that the EEG data differentiates depending on the presented stimulus being a target or non-target, and possibly based on correctness of the subject’s response. Therefore, we tested different data subsets including epochs with the target stimulus (no assumption on the response), epochs with target stimulus and correct response, non-target stimulus (no assumption on the response), and with correct response (no assumption on the stimulus type). Our data indicates that performance is the worst for target stimulus and correct responses combined and the best results were obtained for all available epochs. The classification is performed for conditions L1 and L3 since L0 neither includes the presentation of stimuli nor the recording of responses. Suspecting that those results are due to the data quantity and not its intrinsic properties, we randomly subsampled the larger data subsets to the size of the subset with target stimuli and correct responses. However, the tendency remains the same and overall accuracy drops because of the reduced size of the training set.

##### Leave-One-Out

**Figure 11:**
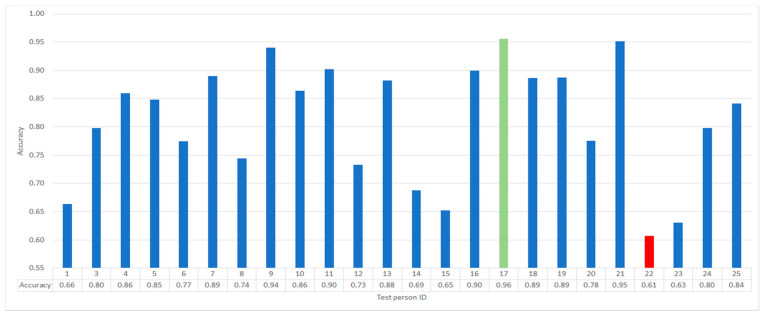
The results of cross validation with a leave-one-out approach. The classifier separates L0 and L3 in the 1.5 days of training data condition. Subject 2 was removed during the initial data preparation stage due to insufficient data quality. The average score was 81.1% and all subjects achieved accuracy above chance level.

For the optimized size of the neural network, we applied a leave-one-out approach. While this approach has limited suitability for the optimization procedure, it can be successfully used for further investigations. In particular, it shows a potential in collecting more data and allows to gain better understanding of the interpersonal data variability. On Figure 11, the results for leaving the subject x (x = 1, 3, ..., 25) out are presented. The resulted average is 81.12%, which is substantially higher than 74.31% on the test set in 16-4-4 subject-wise data division. The highest results on Figure 11 (green) go as high as 96%. In contrast, the lowest result for the subject 22 (red) yields 61%. The variation of the results appears very large.

## 4. Discussion

Technological advances in the last decade have led to considerable changes in the health-care system, offering novel approaches to assessment and treatment. The aim of the present investigation was threefold: we wanted to assess (1) if a mobile EEG setup can be used to track cognitive load in healthy participants, (2) if the different levels of effort can be classified based on machine learning approaches, and (3) the ease of use perceived by the participants. The mobile EEG solution with tablet-based interaction can detect changes in cognitive load as reflected by alterations in different frequency bands. Importantly, an increase in frontal theta and a decrease in occipital alpha was confirmed as robustly observed by clinical and research EEG devices. In addition, the integrated machine learning models could discriminate above chance level and between the most distinct conditions with an accuracy of 86%. A majority of participants indicated good usability and the data quality was at least in an acceptable range. Accordingly, the set-up may be a feasible method to track distinct levels of mental effort or cognitive load.

### 4.1. Behavioral Data

First of all, we investigated the feasibility of the implemented task to induce different levels of mental effort as reflected by the behavioral data. We expected significant differences in the fraction of correct responses as well as mean reaction times reflecting the increasing difficulty level across conditions. Behavioral outcomes largely confirmed different difficulty levels of condition 1–3. Accordingly, reaction times increased across conditions; however, only comparisons between condition 1 (L1) with condition 2 (L2) and 3 (L3) were significant. As a second indicator of task difficulty, the fraction of correct responses (FC) as a measure of accuracy was calculated. All comparisons between conditions were significant. While these results are in line with our expectations, larger differences between conditions (i.e., more distinct difficulty levels) may have facilitated data interpretation of the EEG signal. This is an important aspect that should be considered in upcoming investigations. Based on previous research, we did not integrate a more difficult condition such as a 3-back trial to prevent feelings of frustration and drop-out [7,38]. Instead, we chose the current design as a trade-off between distinct difficulty levels and feasibility for participants.

### 4.2. Cognitive Load & EEG Spectral Power

EEG spectral power can be used as a proxy of cognitive load. In particular, it has been shown that alpha and theta spectral power are meaningful indicators of mental effort [7]. Across lobes and conditions, we observed a drop in the alpha spectral power. However, this drop was only significant for the occipital lobe. In previous studies, decreased occipital alpha activity has been associated with higher cognitive effort [30] such as indicated by an increased level of visual scanning [39]. In addition, Gundel and Wilson [39] detected an increase in frontal theta activity that was associated with mental effort. Our findings are in line with this observation: we reported an increase in theta—and beta—bands across conditions. Previously, an increase in both bands has been associated with higher cognitive load (for review see [40]). In particular, theta spectral power is indicative of the allocation of cognitive resources rendering it a useful predictor of cognitive load [30,40]. In line with our observations, this holds particularly true for frontal regions [41]. Beta has been linked to states of increased alertness and engagement [42]. While it is often located most prominently at occipital and temporal sites, significant effects in our investigation emerged in the parietal lobe. Differences in the observed data pattern may result from specific aspects of the task design. Following this line of interpretation, it has been shown that different tasks used to induce workload result in differences in the data structure [30]. For delta, we observed a continuous increase in the spectral power with higher cognitive load. This is in line with observations linking delta band spectral power to tasks necessitating internal concentration [43]. Accordingly, our data largely confirms results of previous investigations, and thus supports the feasibility of mobile EEG setups to track mental effort.

### 4.3. Classification with Mahine Learning

The applied machine learning models showed promise in the context of potential practical applications to discriminate between cognitive load levels. The obtained results reached an accuracy level of 86% for discrimination between most distant conditions. This is in line with the observations by Hogervorst and colleagues [5]. In parallel to our findings, the authors observed best performance for the most distinct conditions reflecting rest and highest task load.

Exploring different train/test data divisions showed that personal calibration improves the results substantially; however, adding session calibration is the most promising setup. On the other hand, the leave-one-out approach revealed that increasing the training data set further improves the accuracy from 74% (16-4-4 division) to 81% on average. In addition, we observed very high variance in the leave-one-out setup ranging from 61% to 96%. One possible explanation is that the low classification accuracy in some cases results from the higher difficulty of condition 3 (L3) of the N-back task.

Our results are consistent with those reported by Brouwer and colleagues [7]. Their machine learning models were designed to distinguish the 0-back from 2-back conditions. The best performing models resulted in 80–90% range of accuracy, similarly revealing large personal variation and reduced accuracy when conditions were closer together. Despite the design differences, our results are in line with previous investigations and thus support the potential of mobile EEG devices to discriminate cognitive states associated with different levels of cognitive load. Interestingly, filtering data by target or non-target marking and by the response correctness declined the performance of the classifier. The differences may be related to the high variation due to the data set size and should be further investigated.

While our research was focused on the application of mobile EEG sensors for workload quantification and the comparison of the results to preceding investigations with similar task designs, it is important to track the development of the more general machine-learning methods applied to EEG-based workload classification. Our results are consistent with earlier projects such as [44] that used similar methods (e.g., shallow neural networks). Importantly, the accuracy reported for cross-task classification is rather low, which is in line with the high data variability we observed. More recent publications suggest that larger data sets in combination with more complex deep-learning algorithms, such as recurrent and convolutional networks or even their combinations [45,46] may be promising alternatives that need further investigation. Moreover, deep learning algorithms are a promising candidate in terms of handling cross-task classification [47]. In this context, EEG data collected during workload-related task has become openly available [48,49] and hence pave the way for investigations on a larger scale that can be used to evaluate the reproducibility of our findings.

### 4.4. Data Quality

The analysis was conducted using a fully automatic pre-processing pipeline. Following Kutafina et al. [27], as an overall index of data quality, the amount of data rejected on each step of the analysis was recorded. The raw (only filtered and epoched) data set contain 90 epochs of 1500 ms for the baseline measurement (L0), and 150 epochs of the same duration for each task condition (L1–L3). After additional processing steps, approximately 14% of the data acquired on Day 1 and 10% of the data acquired on Day 2 were rejected. The amplitude threshold accounted for approximately two thirds of the rejected data. Our results indicate that the data rejection rate is within an acceptable range.

### 4.5. Usability

Participants indicated on average good usability according to the SUS. In addition, according to the TUI-II, participants showed low levels of anxiety to test the setup and were curious about the technology. While levels of skepticism were low after completion of the cognitive tasks, participants perceived the setup as user-friendly (i.e., easy to understand and use), indicated that they are interested in exploring new technologies, found the setup accessible and expected a benefit from using it for certain tasks. Apart from the quantitative assessment, we also evaluated the device handling from the experimenter perspective. Participants were able to attach the electrodes independently, used the saline solution, and mounted the device correctly. The feedback on the recording software regarding electrode impedance facilitates the process. However, the participants expressed concerns about correct electrode placement, despite a rigid EEG frame. Therefore, attaching the electrodes is a possible draw-back of the setup. It requires good manual precision and can be difficult for EEG-naive participants. This obstacle may be particularly prominent in patients who are elderly or lacking digital literacy. We observed that during the first session, presence and support by the experimenter was beneficial to facilitate handling of the mobile setup. Therefore, a supervised training session should be considered when planning home-based use of the device. Of note, while the rigid plastic construction has many advantages, such as structured electrode positioning, it caused some discomfort in individual participants. This aspect has already been discussed by Ekandem and colleagues [50] who stress the importance of considering the ergonomics of modern low-cost brain computer interfaces—in particular for prolonged use. Our qualitative assessment of user comfort highlights the need to investigate these aspects also for short-duration usage (i.e., ~1 h). Navigation of the tablet, on the other hand, was convenient due to simplistic design and instructions.

### 4.6. Limitations

Generalizability of the data is limited based on the participant selection. All participants were university students; hence, it is likely that they are exposed to technology on a daily basis. Results with regard to usability but also neural activation patterns may differ for a more diverse audience including older individuals or digitally illiterate. While the classification approach was able to discriminate with an accuracy 86% between the most distinct classes, performance may be limited due to the sample size. It has to be determined if, with a larger training set and added personal calibration, the accuracy of discrimination becomes sufficient to provide, for instance, objective assessments of home-based cognitive computer trainings.

## 5. Conclusions

In the current investigation, we tested a fully mobile setup for cognitive training tasks combined with EEG recording to detect cognitive load. The setup was based on a mobile EEG device combined with a tablet to display the cognitive task to allow for easy mounting and performing self-testing. Analysis of the behavioral data confirmed differences in task performance depending on task difficulty. Furthermore, we found a decrease of occipital alpha frequency band power and an increase of frontal theta frequency band power upon higher difficulty of the task confirming our main hypothesis. In addition, automatic cognitive effort classification revealed that the machine learning approach discriminates between the most distinct levels of cognitive load with an accuracy of 86%.

Our findings suggest feasibility of the fully mobile setup to detect distinct levels of cognitive load such as reflected by band power changes. In addition, subjectively-rated usability is adequate with an initial in-person training session of the electrode mounting. Future investigations are needed to evaluate results in more diverse samples including a wider age range and patient groups.

## Figures and Tables

**Table 1:** Descriptive statistics.

	*M*	*SD*	Min	Max	Range
Education	15.8	2.6	13	21	8
Education father *	15.2	3.3	9	18	9
Education mother *	14.7	3.4	9	21	12
Digit span forwards	8.2	1.9	5	12	7
Digit span backwards	6.8	1.4	5	11	6
Verbal intelligence (WST)	102.5	8.1	89	118	29
System Usability Scale (SUS)	79.6	11.2	57.5	95.0	37.5

Notes. *N* = 24. * one missing data point, *n* = 23.

## Data Availability

The data presented in this study are available on request from the corresponding author. The data are not publicly available due to restrictions based on the informed consent statement. The code for EEG data processing and machine learning is available from https://github.com/rwth-imi/mEEG_cognitive_effort_tracking (accessed on 29 July 2021).

## Appendix A

**Table A1:** Statistics on power measures in the frequency bands.

	Baseline Comparisons			Active Comparisons		
	L0–L1	L0–L2	L0–L3	L1–L2	L2–L3	L1–L3
Theta						
Frontal	*t* = −5.8, *p* < 0.001	*t* = −14.3, *p* < 0.001	*t* = −8.4, *p* < 0.001	*t* = −2.5, *p* = 0.02	*t* = −3.0, *p* = 0.007	*t* = −4.7, *p* < 0.001
Temporal	*t* = −1.8, *p* > 0.05	*t* = −1.7, *p* > 0.05	*t* = −2.3, *p* = 0.03	*t* = 0.4, *p* > 0.1	*t* = −1.1, *p* > 0.1	*t* = −0.8, *p* > 0.1
Occipital	*t* = −2.5, *p* = 0.02	*t* = −3.2, *p* = 0.004	*t* = −3.2, *p* = 0.004	*t* = 0.5, *p* > 0.1	*t* = −1.4, *p* > 0.1	*t* = −1.4, *p* > 0.1
Parietal	*t* = −2.0, *p* > 0.05	*t* = −3.8, *p* = 0.001	*t* = −3.1, *p* = *0*.005	*t* = −1.5, *p* > 0.1	*t* = −0.7, *p* > 0.1	*t* = −1.9, *p* > 0.05
Delta						
Frontal	*t* = −5.5, *p* < 0.001	*t* = −10.4, *p* < 0.001	*t* = −8.1, *p* < 0.001	*t* = −3.1, *p* = 0.005	*t* = −2.5, *p* = 0.02	*t* = −4.7, *p* < 0.001
Temporal	*t* = 0.7, *p* > 0.1	*t* = 0.6, *p* > 0.1	*t* = −2.3, *p* = 0.03	*t* = −0.1, *p* < 0.1	*t* = −2.6, *p* = 0.02	*t* = −2.3, *p* = 0.03
Occipital	*t* = −3.1, *p* = 0.005	*t* = −3.9, *p* = 0.001	*t* = −5.9, *p* < 0.001	*t* = −0.7, *p* > 0.1	*t* = −2.3, *p* = 0.03	*t* = −3.0, *p* = 0.006
Parietal	*t* = −1.6, *p* > 0.1	*t* = −3.5, *p* = 0.002	*t* = −4.9, *p* < 0.001	*t* = −1.6, *p* > 0.1	*t* = −1.5, *p* > 0.1	*t* = −2.9, *p* = 0.01
Alpha						
Frontal	*t* = 0.8, *p* > 0.1	*t* = 1.4, *p* > 0.1	*t* = 1.4, *p* > 0.1	*t* = 0.5, *p* > 0.1	*t* = 0.8, *p* > 0.1	*t* = 1.1, *p* > 0.1
Temporal	*t* = 1.8, *p* > 0.05	*t* = 2.2, *p* = 0.04	*t* = 1.6, *p* > 0.1	*t* = 0.8, *p* > 0.1	*t* = 0.1, *p* > 0.1	*t* = 0.6, *p* > 0.1
Occipital	*t* = 0.8, *p* > 0.1	*t* = 2.7, *p* = 0.01	*t* = 2.5, *p* = 0.02	*t* = 3.1, *p* = 0.005	*t* = 1.5, *p* > 0.1	*t* = 3.0, *p* = 0.006
Parietal	*t* = 0.3, *p* > 0.1	*t* = 0.5, *p* > 0.1	*t* = 1.1, *p* > 0.1	*t* = 0.2, *p* > 0.1	*t* = 1.0, *p* > 0.1	*t* = 1.1, *p* > 0.1
Beta						
Frontal	*t* = −0.7, *p* > 0.1	*t* = −0.9, *p* > 0.1	*t* = −1.2, *p* > 0.1	*t* = −0.4, *p* > 0.1	*t* = −0.5, *p* > 0.1	*t* = −0.9, *p* > 0.1
Temporal	*t* = 0.1, *p* > 0.1	*t* = −0.3, *p* > 0.1	*t* = −2.3, *p* = 0.03	*t* = −0.3, *p* > 0.1	*t* = −2.1, *p* = 0.05	*t* = −2.1, *p* = 0.05
Occipital	*t* = −0.5, *p* > 0.1	*t* = −0.4, *p* > 0.1	*t* = −2.6, *p* = 0.02	*t* = −0.0, *p* > 0.1	*t* = −1.9, *p* > 0.05	*t* = −2.3, *p* = 0.03
Parietal	*t* = 0.6, *p* > 0.1	*t* = −1.5, *p* > 0.1	*t* = −4.9, *p* < 0.001	*t* = −1.8, *p* > 0.1	*t* = −2.7, *p* = 0.01	*t* = −4.2, *p* < 0.001
